# Emotional expressions evoke a differential response in the fusiform face area

**DOI:** 10.3389/fnhum.2013.00692

**Published:** 2013-10-28

**Authors:** Bronson Harry, Mark A. Williams, Chris Davis, Jeesun Kim

**Affiliations:** ^1^School of Psychology, Bangor UniversityBangor, UK; ^2^Department of Cognitive Science, Macquarie University, SydneyNSW, Australia; ^3^The MARCS Institute, University of Western Sydney, SydneyNSW, Australia

**Keywords:** emotion, face processing, multivariate pattern analysis, fusiform face area

## Abstract

It is widely assumed that the fusiform face area (FFA), a brain region specialized for face perception, is not involved in processing emotional expressions. This assumption is based on the proposition that the FFA is involved in face identification and only processes features that are invariant across changes due to head movements, speaking and expressing emotions. The present study tested this proposition by examining whether the response in the human FFA varies across emotional expressions with functional magnetic resonance imaging and brain decoding analysis techniques (*n* = 11). A one vs. all classification analysis showed that most emotional expressions that participants perceived could be reliably predicted from the neural pattern of activity in left and the right FFA, suggesting that the perception of different emotional expressions recruit partially non-overlapping neural mechanisms. In addition, emotional expressions could also be decoded from the pattern of activity in the early visual cortex (EVC), indicating that retinotopic cortex also shows a differential response to emotional expressions. These results cast doubt on the idea that the FFA is involved in expression invariant face processing, and instead indicate that emotional expressions evoke partially de-correlated signals throughout occipital and posterior temporal cortex.

## Introduction

Many functional models of face processing propose that the neural pathways involved in face identification are separate from the pathways that are involved in recognizing emotional expressions (Haxby et al., 2000; O'Toole et al., 2002; for a detailed review see, Calder and Young, 2005). A proposal common to these models is that the fusiform face area (FFA), a face selective brain region located in the ventral temporal lobes (Kanwisher and Yovel, 2006), is involved in face identification and as such, only processes features that are invariant across changes due to head movements, speaking and emotional expressions. Recognizing emotional expressions on the other hand, is thought to involve other brain regions such as the superior temporal sulcus and the amygdala (Ishai, 2008).

Positing a dichotomy between the processing of invariant and changeable properties of faces (and linking to different anatomic substrates) has been described as the standard view in the area (Calder and Young, 2005; Cohen Kadosh et al., 2010). Recently, this proposed dichotomy has received support from a number of studies that have used multivariate pattern analysis (MVPA) to determine whether changeable or non-changeable features evoke a differential response within face selective brain regions. This analysis technique measures the distributed patterns of activity evoked in a brain region to determine whether different perceptual states are associated with distinct patterns of activity (Normam et al., 2006; Williams et al., 2007). Consistent with the dichotomous view, these studies showed that changeable features, such as head or gaze orientation, evoke distinct patterns of activity in face selective regions located in the STS (Carlin et al., 2011), whereas non-changeable features (such as identity) evoke distinct patterns of activity in the FFA and other regions in the ventral temporal lobes (Kriegeskorte et al., 2007; Op de Beeck et al., 2010; Nestor et al., 2011).

Recently, this proposed anatomical dichotomy has been challenged by evidence that the FFA is involved in processing emotional expressions (Ganel et al., 2005; Fox et al., 2009a; Xu and Biederman, 2010). These studies reported repetition suppression effects in the FFA to repeated presentations of the same emotion, suggesting that this region contains neurons that are selective for different facial expressions. However, the index of information processing (fMRI adaption) used in these studies can be affected by attention-dependent expectation (e.g., Summerfield et al., 2011; Larsson and Smith, 2012) and possibly by task demands (Cohen Kadosh et al., 2010). Given that results from this measure may be difficult to interpret (due to the different factors that can influence it), the present study aimed to determine whether the FFA shows a differential response to emotional expressions using a measure that more directly assesses the information processed by a brain region (i.e., MVPA). The use of MVPA also avoided possible repetition related effects associated with fMRI adaption.

The aim of MVPA is to investigate whether different perceptual states evoke distinct patterns of activity within a brain region. Unlike standard univariate analysis, MVPA does not involve averaging over voxel intensities, but instead this technique examines the fine spatial patterns across voxels that are associated with different stimuli. Most MVPA studies make use of machine learning techniques in order to index whether response patterns contain reliable information about stimulation conditions. This analysis involves training a pattern classifier to discriminate the patterns of neural activity evoked when viewing different categories of stimuli. Broadly, the classifier learns a discriminant function for each category, which consists of a set of parameters (one for each voxel) that minimizes the prediction error (e.g., cost function, sum of squares) for a labeled dataset. Prediction involves calculating a weighted sum of input activation patterns from the learned parameters for each discriminant function. Typically, the predicted class corresponds to the discriminant function with the highest weighted sum. If the classifier can predict the category of stimulation from the activation patterns (at above chance levels), then this indicates that the brain region shows a distinct pattern of response to the different categories of stimuli. However, since classifiers are prone to over-fitting, classification performance can be over-estimated if the training data is used to evaluate the classifier. An unbiased estimate of classifier performance is obtained by training and testing the classifier on different sets of data.

Our rationale for the present study is that if a classifier was able to accurately predict expressions from the pattern of activity within the FFA, then this would indicate that different emotional expressions evoke distinct patterns of activity in this region and so suggest that the face processing in the FFA codes emotional expression information.

## Methods

### Participants

Twelve graduate students gave fully informed consent to participate in the study (*M* = 25 years, 10 males). One participant that moved more than 4 mm across the complete scanning session was removed from the analysis. All participants were unaware of the aim of the study. The research was approved by the University of Western Sydney Human Ethics Committee and adhered to the principles contained within the Declaration of Helsinki.

### Materials

Images of 16 young adult, Asian and Caucasian individuals depicting fear, anger, happiness, disgust, and sadness, as well as a neutral pose, were selected from the NimStim face database (Tottenham et al., 2002). Images of the same individuals were selected for all emotions, and the set was composed of the same number of male and female models. The face region in these images was cropped and the contrast and luminance standardized in Photoshop. Sixteen images of houses were also selected from the internet and prepared in a similar manner as the face stimuli.

### Procedure

Participants completed two types of scanning run; a localizer run and an emotion run. The purpose of the localizer run was to collect an independent dataset to localize the left and right FFA in each participant. Each localizer run comprised 21, 18-s blocks. Blocks 1, 6, 11, 16, and 21 were a fixation-only rest condition. All remaining blocks consisted of either fearful face or house stimulation. Face and house blocks alternated and block order was counter balanced across runs. The emotion runs comprised 15, 18-s blocks per run. All odd numbered blocks were fixation-only rest blocks, and all even blocks consisted of faces all expressing the same emotion (neutral, fear, happiness, anger, disgust or sadness). Blocks of house images were also included to verify that the classifier could discriminate the patterns elicited by objects from different categories (i.e., faces vs. houses). Each category of stimulus appeared once per run and order of conditions was randomized across runs.

Each block of stimulation (for both type of run) involved presenting the 16 face or house stimuli once in a random order. Each stimulus was presented for 500 ms followed by a 500 ms blank interval between stimuli. Two of the stimuli in each block were randomly selected to appear in consecutive trials. Participants were instructed to indicate detection of these repetitions by pressing a button. Participants completed two localizer runs, followed by 8–12 emotion runs.

### fMRI acquisition

Brain images were acquired in a Phillips Achieva 3.0T TX scanner with an 8 channel head coil. Functional images were collected with a T2^*^ weighted, gradient echo planar imaging sequence (repetition time = 3000 ms; echo time = 32 ms; flip angle = 90°; field of view = 240 mm × 240 mm; acquisition matrix = 160 × 160; in plane resolution 1.5 mm × 1.5 mm; slice thickness 2 mm; 20% slice gap). Volumes consisted of 26 slices covering the ventral temporal and frontal lobes that were angled to minimize coverage of the sinus cavity. High resolution T1 weighted anatomical images was also acquired for each participant (3D-MPRAGE sequence; voxel size = 1 mm isotropic; field of view = 240 mm × 240 mm; repetition time = 2110 ms; echo time = 3.54 ms; flip angle = 9°).

### Image processing and analysis

Image pre-processing and localization of the regions of interest was performed in SPM8 (Welcome Department of Imaging Neuroscience; http://www.fil.ion.ucl.ac.uk/spm/software/spm8/). The first four volumes of each run were automatically discarded before image processing. Images from the localizer and emotion runs were coregistered by aligning and unwarping all images to the first volume. Images from the localizer run were spatially smoothed with a 6 mm Gaussian filter, and the time series of each voxel was high pass filtered at 1/128 Hz. Correlations between scans and periods of face and house stimulation were modeled by a standard hemodynamic response function at each voxel. A *t*-test examining greater activity during face stimulation vs. house stimulation was used to localize the right and left FFA (*p* < 0.001 uncorrected). Additional spherical masks with the same volume as the FFA masks were also created for the purpose of a control analysis (see Results). These masks were centered on the left parahippocampal place area (lPPA), a region that selectively responds to images of scenes (Epstein et al., 1999), and the early visual cortex (EVC). The PPA mask was centered on the peak voxel in the parahippocampal gyrus for the *t*-test examining greater activity during house stimulation vs. face stimulation in the localizer runs (*p* < 0.001 uncorrected). Parahippocampal gyrus was identified as the most medial gyrus in the ventral temporal lobes, and the fusiform was identified as the gyrus lateral to the parahippcampal gyrus. Anatomical scans were used to construct the EVC masks by placing the spherical mask to cover as much of the occipital pole as possible. Face selective regions in the STS were not examined in the present study since the slice prescription did not cover the dorsal temporal lobes.

Statistical images (*T*-maps) were spatially normalized into MNI space for the purposes of reporting the coordinates of the peak voxels. All the remaining analysis was conducted in native space to preserve the fine patterns of activity.

Pattern analysis was conducted on the unsmoothed, realigned data from the emotion runs with the Princeton Multi-Voxel Pattern Analysis toolbox (The Princeton Neuroscience Institute; http://code.google.com/p/princeton-mvpa-toolbox/). The time-course data for all voxels within a region of interest was extracted. The time-course for each individual voxel was z-score transformed so that the mean and standard deviation of the signal across each run was zero and one (respectively). This transformation was applied independently to each voxel within a ROI to ensure that highly variable voxels did not bias the classifier, and to facilitate optimization of the classifier weights during training. Normalization was performed separately for each run. Time points within each block of stimulation were averaged to produce one pattern of activity for each condition in each run. To account for hemodynamic lag, only time points corresponding to data collected 9–18 s after block onset were averaged.

To examine whether each emotional expression evoked a distinct pattern of activity in the regions of interest, a single class logistic regression classifier was trained to distinguish each emotional expression from all other expressions with the block averaged data from the emotion runs. Classifier performance was evaluated with a leave one run out cross validation procedure. This involved training a single class logistic regression classifier to learn a mapping between the block averaged patterns and the corresponding class labels (active conditions) for all but one run, and then using the trained classifier to predict the category of stimuli from the test patterns in the remaining run. The cross validation procedure was repeated until reach run was used as the test set, and classification performance was averaged over all iterations.

Prior to classifier training, a simple feature selection procedure was used to reject any voxel that did not show a significantly different response across the categories of stimuli. This involved entering the activation data for each voxel from all of the training patterns into a one way ANOVA with stimulus category as different levels of the factor. Any voxel that did not pass a liberal threshold (*p* < 0.05) were not included in training or testing. Evaluating classifier performance with a cross validation procedure ensured that the classifier was tested on data that was independent from the data used for feature selection (i.e., localization and ANOVA feature selection) and classifier training. Thus, this procedure provides a measure of classifier performance that is free from overfitting the dataset and so avoiding circular analysis.

This procedure was repeated for the sphere over the EVC and bilateral PPA to determine whether a differential response to emotional expressions was also present in early visual and scene selective processing regions.

## Results

### Behavioral performance

Average one-back performance was high, with participants detecting 92.4% of all stimulus repetitions. A repeated measures analysis did not reveal any significant differences across the different categories of stimuli [*F*_(6, 5)_ = 1.3, *p* = 0.39].

### Classification analysis

Classification performance for each region and emotional expression was entered into a 5 (Region) × 6 (Expression) repeated measures ANOVA to examine whether there were any differences in the pattern of classification across the regions of interest. This analysis showed that there was a significant main effect of region [*F*_(4, 40)_ = 9.0, *p* < 0.001]. The main effect of expression [*F*_(5, 50)_ = 1.05, *p* = 0.4] and the interaction between region and expression was not significant [*F*_(20, 200)_ = 0.68, *p* = 0.84]. Classification performance was averaged over emotional expression for each participant and ROI to examine the main effect of region. Two-tailed, paired sample *t*-tests revealed that left FFA classification performance was higher than the left PPA [*t*_(10)_ = 2.84, *p* = 0.012] but not the EVC [*t*_(10)_ = −0.874, *p* = 0.403]. Similarly, right FFA classification performance was higher than the right PPA [*t*_(10)_ = 4.565, *p* = 0.001] but not the EVC [*t*_(10)_ = −0.833, *p* = 0.424].

Classification analysis showed that most emotional expressions could be decoded from the patterns of activity in the right (mean MNI coordinates *x* 42, *y* −47, *z* −22) and left FFA (mean MNI coordinates *x* −40, *y* 47, *z* − 23). Table 1 shows classification performance for each emotional expression and the *p*-value for a single sample *t*-test (two tailed) comparing classifier performance against chance (50%). For a Bonnferoni corrected criteria (α = 0.0083) the results showed that all emotional expressions could be decoded in the right FFA, and all but happiness and anger could be decoded in the left FFA. These results indicate that most emotional expressions evoke partially non-overlapping mechanisms in the FFA. Classification performance in the EVC showed a similar pattern as the fusiform, with only fear failing to be classified more accurately than chance. Classification performance in left and right PPA was not significantly above chance for all emotional expressions.

**Table 1:** **Classifier performance for one vs. all classification in the right and left FFA and extra-striate**.

	**Early visual cortex**		**Left FFA**		**Right FFA**		**Left PPA**		**Right PPA**	
	**Average performance**	***p***	**Average performance**	***p***	**Average performance**	***p***	**Average performance**	***p***	**Average**	***p***
Neutral	0.583	0.0075	0.602	0.0019	0.557	0.0040	0.549	0.0925	0.506	0.4136
Happiness	0.594	<0.0001	0.533	0.0469	0.581	0.0008	0.484	0.7205	0.515	0.2911
Fear	0.557	0.0245	0.569	0.0010	0.572	0.0011	0.497	0.5342	0.525	0.1560
Anger	0.566	0.0019	0.531	0.0758	0.551	0.0002	0.512	0.2892	0.526	0.2104
Disgust	0.555	0.0004	0.564	<0.0001	0.558	0.0071	0.497	0.6082	0.509	0.4039
Sadness	0.583	<0.0001	0.585	0.0002	0.560	0.0058	0.572	0.0103	0.550	0.0146

## Discussion

The present study showed that emotional expressions could be decoded from the pattern of activity in the FFA. Contrary to many models of face processing (Haxby et al., 2000; O'Toole et al., 2002; Winston et al., 2004), these findings support claims that the FFA is involved in processing emotional expressions (Ganel et al., 2005; Tsuchiya et al., 2008; Fox et al., 2009a; Xu and Biederman, 2010; Kawasaki et al., 2012) and is consistent with evidence that emotion selective neurons are distributed throughout the ventral temporal lobes (Hadj-Bouziane et al., 2008; Morin et al., 2010). Although classification accuracy in the FFA was low, the present findings are similar with previous studies examining the patterns evoked by other within-category distinctions with MVPA (Eger et al., 2008; Op de Beeck et al., 2010). For example, Op de Beeck et al. (2010) showed that it was possible to decode whether blocks of faces consisted of infant or elderly individuals with approximately 60% performance in the FFA. The findings of the pattern analysis in the EVC also showed that most emotional expressions could be distinguished from all other expressions, indicating that emotions evoke distinct patterns of activity throughout the visual cortex. Indeed, classifier performance did not change across the EVC and FFA, suggesting that the FFA is just as sensitive to information related to emotional expressions as the early visual system. This finding contradicts the view that the FFA is involved in expression invariant processing, and is consistent with evidence that the FFA is involved in processing identity preserving transformations, such as view (Xu et al., 2009; Kietzmann et al., 2012).

Evidence that the FFA is involved in processing changeable facial features raises the question of what role this region plays in the face processing network. It is possible that the FFA is functionally specialized to extract expression invariant face information but shows a differential response to emotions because this region occupies an intermediate stage in the face identification pathway (e.g., Rotshtein et al., 2005). Face selective brain regions are found throughout the posterior and anterior fusiform (Allison et al., 1999; Puce et al., 1999; Tsao et al., 2008; Rajimehr et al., 2009; Ku et al., 2011; Nsar and Tootell, 2012; Tanji et al., 2012), suggesting that visual analysis of faces continues beyond the FFA. Indeed, studies using MVPA to examine patterns evoked by individual faces have shown that face exemplar patterns are present in the anterior temporal lobes (Kriegeskorte et al., 2007; Nestor et al., 2011). Moreover, the face identification deficits associated with prosopagnosia are related to disruption of the connections between the posterior and anterior temporal lobes (Avidan and Behrmann, 2009; Thomas et al., 2009). So, one possibility might be that the FFA is a part of a ventral pathway that extracts identity related information (c.f., Haxby et al., 2000) and that the differential response to emotional expressions observed in the present study indicates that expression invariant representations might be fully formed only in the anterior face processing regions.

An alternative proposal to the one described above is that the FFA contributes to both identification and emotion processing because this region is involved in processing the configural information associated with invariant and changeable facial features. There is considerable evidence that the FFA is involved in processing the configuration of facial features (Yovel and Kanwisher, 2004; Schiltz and Rossion, 2006; Liu et al., 2009; Harris and Aguirre, 2010). Given that emotion recognition relies on the perception of configural information (McKelvie, 1995; Calder et al., 2000b; Durand et al., 2007), it is plausible that this region processes the configuration of changeable facial features. This suggestion is in line with recent evidence (Cohen Kadosh et al., 2010) showing that the extent to which a face processing task relies on configural information (e.g., identification and emotion recognition vs. gaze direction) has a stronger effect on FFA activity compared to changes in stimulus content (e.g., identity or expression). However, evidence that the FFA processes changeable features does not imply that this region explicitly represents facial motion or even emotional expressions. Face processing regions in the STS show a much larger increase in activity for moving compared to static faces (Fox et al., 2009b; Pitcher et al., 2011) and deficits in emotion recognition are typically associated with damage to regions involved in emotion processing such as the amygdala and the insula (Adolphs et al., 1994; Calder et al., 2000a). It is more likely that the FFA processes static form-based information that is utilized by other brain regions depending on task demands. That is, judgments about identity and emotional expressions may both rely on information represented in the FFA, but recruit different processing pathways in the extended face processing network (Ishai, 2008; Atkinson and Adolphs, 2011; Said et al., 2011).

The results of the present study may provide clues about which of these two accounts best explain the observed differential response to emotional expressions in the FFA. If the FFA was a part of a pathway that gradually extracts expression invariant information, then it should be the case that the FFA is less sensitive to emotion information than the EVC. However, the present study did not reveal any differences in classification performance for the FFA and EVC. So it might be possible that the FFA is involved in processing both identity and emotional expressions. Of course, to draw this conclusion it would also need to be shown that emotion and identity information are decoded at similar levels of accuracy in the FFA for stimuli that have been matched for visual similarity. If, on the other hand, such an experiment revealed that identity information evoked more distinct patterns than emotional expressions, then this would support the view that the FFA is partially invariant to emotion information.

The finding that emotional expressions could also be distinguished from the patterns of activity in the visual cortex, indicates that emotional expressions evoke activity in partially non-overlapping mechanisms in the earliest stages of visual processing. Since EVC forms an image-like representation of incoming visual input, evidence of partially de-correlated signals in retinotopic cortex suggests that emotional expressions are somewhat visually distinct. This finding is consistent with the idea that emotional expressions have evolved to minimize the overlap between different expressions and so facilitate transmission of emotional information to observers (Smith et al., 2005; Susskind et al., 2008). Although classification results in EVC and face selective regions were similar, it is likely that the differential response to emotional expressions in the EVC is based on retinotopic registration of diagnostic local features (Petro et al., 2013) whereas in the temporal lobes is it based on the activity of neurons that are responsive to the configural properties associated with the different emotional expressions.

In conclusion, the present study found that emotional expressions evoke distinct patterns of activity throughout the occipito-temporal cortex, including the FFA. This finding runs against the widespread view that the FFA is involved in expression invariant processing. Indeed, the results of the one vs. all classification analysis showed that emotional expressions evoke partially separable neural mechanisms across early vision, suggesting that emotional expressions have evolved to support efficient signal decoding in the brain.

### Conflict of interest statement

The authors declare that the research was conducted in the absence of any commercial or financial relationships that could be construed as a potential conflict of interest.
